# Efficacy and safety of microfragmented adipose tissue versus platelet-rich plasma, hyaluronic acid and saline for knee osteoarthritis

**DOI:** 10.3389/fmed.2026.1901713

**Published:** 2026-08-11

**Authors:** Di Zhao, Shan-Chuang Chen, Zi-Tao Liu, Ze-Xin Huang, Xiu-Jun Mai, Nan-jun Xu, Jun Liu, Tao Jiang

**Affiliations:** 1 Department of Orthopedics, The Second Affiliated Hospital of Guangzhou University of Chinese Medicine, Guangzhou, China; 2 Bone and Joint Research Team of Degeneration and Injury, Guangdong Provincial Academy of Chinese Medical Sciences, Guangzhou, China; 3 Guangdong Second Traditional Chinese Medicine Hospital (Guangdong Province Enginering Technology Research Institute of Traditional Chinese Medicine), Guangzhou, China; 4 The Fifth Clinical Medical College of Guangzhou University of Chinese Medicine, Guangzhou, China

**Keywords:** hyaluronic acid, intra-articular injection, knee osteoarthritis (KOA), microfragmented adipose tissue (MFAT), platelet-rich plasma (PRP) injection

## Abstract

**Purpose:**

To compare the efficacy and safety of intra-articular microfragmented adipose tissue (MFAT) injection with platelet-rich plasma (PRP), hyaluronic acid (HA), and saline injection for knee osteoarthritis treatment.

**Methods:**

PubMed, Embase, the Cochrane Library and Web of Science were searched for randomized controlled trials (RCTs) published before April 15, 2026. Patients were assessed for efficacy (Western Ontario and McMaster Universities Osteoarthritis Index (WOMAC)) score, Knee Injury and Osteoarthritis Outcome Score (KOOS), and visual analog scale (VAS), International Knee Documentation Committee (IKDC), and Tegner scores and safety (treatment-related adverse events (AEs)) outcomes.

**Results:**

Eleven RCTs were identified. At the 6-month and 12-month follow-ups, compared with the control interventions (PRP, HA and saline), intra-articular MFAT injection significantly improved efficacy outcomes (VAS and KOOS symptom). At the 24-month follow-up, intra-articular MFAT injection was significantly more advantageous than control interventions for reducing pain (VAS score) and improving WOMAC scores. Subgroup analysis results of only RCTs directly comparing MFAT with PRP or HA injection were consistent with the primary analysis results: MFAT was significantly superior to PRP and HA in terms of patient-reported outcomes at the 6- and 12-month follow-ups. Heterogeneity was markedly reduced in this subgroup analysis. Regarding safety, compared with control interventions, IA MFAT was associated with no statistically significant difference in AE incidence. The overall methodological quality of the included studies was relatively high.

**Conclusion:**

At both 6- and 12-month follow-ups, intra-articular MFAT significantly improved core symptom endpoints (VAS, KOOS symptom, WOMAC (6- and 24-month)) versus controls (PRP, HA, or saline), whereas distal functional endpoints (IKDC, KOOS-ADL, KOOS-Sport) showed no significant differences. However, all pooled effect sizes remained below MCID thresholds, indicating that current 12-month evidence does not support definitive clinical meaningfulness. Treatment-related AEs were comparable to controls, supporting favorable short-term safety. These hypothesis-generating findings underscore the urgent need for large-scale RCTs with follow-up exceeding 24 months and prespecified subgroup analyses to determine sustained efficacy, disease modification, and late-onset safety.

## Introduction

Knee osteoarthritis (KOA) is a prevalent, age-associated degenerative joint disorder characterized by progressive cartilage degradation, synovial inflammation, and subchondral bone remodeling, contributing to chronic pain, functional impairment, and disability worldwide. In end-stage disease, total knee arthroplasty remains the primary surgical intervention (1); thus, slowing structural deterioration to delay or avoid arthroplasty represents a central therapeutic objective. The Osteoarthritis Research Society International guidelines endorse a multimodal conservative approach, including weight management, exercise, anti-inflammatory agents, and intra-articular HA or corticosteroids (2). In recent years, orthobiologics—autologous biological preparations that modulate the joint microenvironment to support tissue homeostasis and repair—have emerged as a promising paradigm. PRP, the most extensively studied orthobiologic, has demonstrated superior and sustained improvements in pain and function versus placebo, corticosteroids, and HA, mediated by localized release of growth factors and immunomodulatory cytokines (3, 4). With accumulating evidence for PRP, adipose-derived biologics are gaining attention. Adipose tissue offers abundant availability, minimally invasive procurement, high progenitor cell yield, and low immunogenicity (5). Standardized point-of-care mechanical processing now enables MFAT preparation without culture or enzymatic digestion, preserving the stromal vascular fraction, mature adipocytes, microvascular networks, and mesenchymal stromal cells, along with natural tissue architecture and paracrine functions (6, 7). MFAT is enriched with regenerative and immunomodulatory factors, underpinning its chondroprotective, anti-inflammatory, angiogenic, and matrix-remodeling activities (8).

The regulatory landscape for MFAT varies across countries. In the EU, MFAT is classified as an ATMP when substantially manipulated, whereas mechanical microfragmentation may fall outside this regulation in some member states (9). In the US, minimally manipulated autologous MFAT is regulated under Section 361 of the PHS Act, exempt from premarket approval for homologous use, whereas more extensive processing would trigger Section 351 requirements (10). Japan has adopted a permissive conditional early approval system under its Act on the Safety of Regenerative Medicine (11), while China regulates it primarily as a medical device or biological product with rigorous trial requirements (12). This regulatory discordance complicates cross-trial comparability, limits generalizability of existing evidence, and underscores the need for internationally harmonized frameworks to guide MFAT research in KOA.

However, robust, high-level evidence remains insufficient to determine whether MFAT provides clinically meaningful advantages in efficacy or safety over established intra-articular therapies—including HA, PRP, and saline placebo—in KOA patients. To bridge this evidence gap, we conducted a systematic review and meta-analysis of RCTs rated as level I or II evidence. The primary objective was to quantitatively compare MFAT against HA, PRP, and saline across three prespecified endpoints—pain intensity, functional status, and treatment-emergent adverse events—providing an evidence-based appraisal of its comparative benefit–risk profile. Corticosteroid injections were explicitly excluded from the comparator arms due to well-documented concerns: long-term or repeated use is associated with accelerated cartilage catabolism, progressive joint space narrowing, and systemic risks including hyperglycemia and adrenal suppression (13). Thus, we focused the analysis on regenerative interventions—MFAT, PRP, and HA—that possess biologically substantiated tissue-regenerative capacity and a more favorable long-term safety profile, thereby generating clinically actionable evidence to inform clinical decision-making and future guideline iterations.

## Methods

This systematic review was conducted and reported in accordance with the Preferred Reporting Items for Systematic Reviews and Meta-analyses (PRISMA) recommendations (14).

### Search strategy and selection criteria

PubMed, Embase, the Cochrane Library and Web of Science were comprehensively searched from the inception of the database to April 15, 2026, for English studies using the following keywords: “adipose,” “microfragmented,” “adipose tissue,” “platelet rich plasma,” “PRP,” “hyaluronic acid,” “HA,” and “osteoarthritis.” Additionally, the reference lists of all the included RCTs and relevant systematic reviews were hand-searched to identify potentially eligible studies missed by the electronic search; all the candidate articles underwent independent dual screening by two reviewers. Discrepancies in eligibility assessment were resolved through consensus discussion or adjudication by a third reviewer, with documentation of the rationale for all exclusions.

### Eligibility criteria

The inclusion criteria were as follows: (1) adult patients diagnosed with knee osteoarthritis; (2) intervention: the experimental group was treated with MFAT injection, and the control group was treated with PRP, HA or saline injection; (3) outcomes included at least the following outcome measures: Western Ontario and McMaster Universities Osteoarthritis Index (WOMAC) score, Knee Injury and Osteoarthritis Outcome Score (KOOS), visual analog scale (VAS) score (0–100), International Knee Documentation Committee (IKDC) score, Tegner score and treatment-related adverse events (treatment-related AEs); and (4) RCT (only study type included in our analysis).

The exclusion criteria were as follows: (1) patients with prior intra-articular administration of any comparator intervention before enrollment; (2) studies reporting insufficient outcome data to permit quantitative synthesis; and (3) nonrandomized studies, conference abstracts, and meeting proceedings.

### Study selection and data extraction

In accordance with the prespecified eligibility criteria, two independent reviewers conducted duplicate screenings of titles, abstracts, and full texts to identify studies meeting all the inclusion requirements. Discrepancies at each screening stage were resolved through consensus discussion; unresolved disagreements were resolved through formal adjudication by a third senior reviewer. Data extraction was performed independently by the same two reviewers using a standardized, piloted form. The extracted items included the first author, publication year, total sample size, sex distribution, mean age, level of evidence, follow-up duration, intervention details (agent, concentration, volume, and injection frequency), and Kellgren-Lawrence grade. All discrepancies during extraction were resolved via consensus or, if persistent, by arbitration from the third reviewer, with documentation of the rationale for all decisions.

### Risk of bias assessment

The methodological quality of the included randomized controlled trials was assessed using the Cochrane risk of bias tool, which evaluates seven prespecified domains: random sequence generation, allocation concealment, blinding of participants and personnel, blinding of outcome assessment, incomplete outcome data, selective reporting and other bias. Each domain was rated as “low risk,” “unclear,” or “high risk” of bias, with explicit justification documented for all judgments.

### Data synthesis and statistical analysis

Statistical analyses were conducted using RevMan (v5.4; Cochrane Informatics and Knowledge Management Department) for meta-analysis. For continuous efficacy outcomes—including VAS score, KOOS subscales (pain, symptoms, ADL, sport/recreation, and quality of life), and Tegner, IKDC, and WOMAC total scores—we extracted mean baseline-to-endpoint changes with corresponding standard deviations (SD) and computed mean differences (MDs) with 95% confidence intervals (CIs) using inverse-variance weighting. For dichotomous safety outcomes, we calculated risk ratios (RRs) with 95% CIs using the Mantel–Haenszel method. Only outcomes reported in at least two eligible studies were synthesized quantitatively and presented in the main review results, as pooling from a single study precludes estimation of between-study variability and yields no meaningful heterogeneity or summary effect. Heterogeneity was formally assessed using the Cochran Q test and the I^2^ statistic: 0–50% indicated low heterogeneity, >50–80% indicated moderate heterogeneity, and >80% indicated substantial heterogeneity. A random-effects model was applied when I^2^ > 50%; otherwise, a fixed-effects model (inverse variance) was used. Publication bias was evaluated via Egger’s linear regression test for outcomes with ≥10 studies; funnel plot asymmetry was interpreted alongside the statistical test results. Statistical significance was defined as *p* < 0.05 for all hypothesis tests.

## Results

### Selection of included studies

A total of 543 records were identified through the database searches. After removing duplicates, 192 records underwent title and abstract screening, yielding 22 potentially eligible studies. Full-text assessment was then performed, resulting in 11 studies meeting all prespecified inclusion and exclusion criteria. The complete study selection process is summarized in the PRISMA flow diagram (Supplementary Figure 1).

### Characteristics of the included studies

This meta-analysis included 11 RCTs (15–25) enrolling a total of 992 patients with knee OA. With respect to patient eligibility, four RCTs enrolled participants across the full K-L spectrum (grades I–IV), two restricted enrollment to K-L grades I–II, four restricted enrollment to K-L grades II–III, and one restricted enrollment to K-L grades III–IV. Two RCTs assessed outcomes at the 6-month time point, four assessed outcomes at the 12-month time point, and five assessed outcomes at the 24-month time point. Three RCTs utilized leukocyte-rich platelet-rich plasma (LR-PRP), three used leukocyte-poor platelet-rich plasma (LP-PRP), and all the studies applied standardized mechanical processing of adipose tissue using the Lipogems system. Trial characteristics are summarized in Table 1. With respect to time-point-specific data extraction, Baria et al.’s primary study contributed data to the 6-month analysis, Dallo et al.’s study informed the 12-month analysis, Baria et al.’s extended follow-up study was used exclusively for the 12-month endpoint to avoid overlap with the primary analysis, and Gobbi et al.’s extended follow-up study provided the sole source of 24-month outcome data. This time-point-stratified, nonoverlapping data allocation strategy ensured analytical independence across endpoints, eliminated the risk of duplicate participant inclusion, and preserved statistical integrity and interpretability for each prespecified follow-up interval.

**Table 1:** Characteristics of the included studies.

Study (Year)	Interventions	No. of subjects	Sex (female)	Mean age (y)	K-L grade^a^	Follow-up (mo)
Barfod (2025) (8)	MFAT, Lipogems, 7 ml Saline, 10 ml	60/60	36/34	52.9/51.5	II, III	24
Baria (2022) (17)	MFAT, Lipogems, 7.92 ± 3.87 mL LR-PRP, 5.12 ± 1.12 mL, Angel cPRP system (Arthrex)	28/30	20/10	56.1/51.9	I, II, III, IV	6
Baria (2024) (16)	MFAT, Lipogems, 8.2 ± 3.9 mL LR-PRP, 5.2 ± 1.0 mL, Angel cPRP system (Arthrex)	26/23	18/7	56.7/52.8	I, II, III, IV	12
Dallo (2021) (18)	MFAT, Lipogems LP-PRP+HA, Manual, 3 mL of PRP for every 2 mL of HA, 3 injections, 4 weeks apart	40/40	16/11	61.5/62.5	I, II	12
Gobbi (2023) (19)	MFAT, Lipogems, LP-PRP+HA, Manual, 3 mL of PRP for every 2 mL of HA, 3 injections, 4 weeks apart	40/40	23/18	62.8/62.0	I, II	24
Kaszyński (2022) (20)	MFAT, Lipogems LP-PRP, Manual, 3 injections, 2 weeks apart	20/20	//^b^	55.0/57.0	II, III	12
Molnar (2025) (21)	MFAT, Lipogems, 7 ml HA, Hyalubrix 60^®^, 60mg	35/18	26/14	53.9/58.7	II, III	12
Richter (2024) (22)	MFAT, Lipogems, 7 ml Saline, 7 ml	23/22	8/15	62.6/61.2	I, II, III, IV	12
Ulivi (2023) (23)	Arthroscopic debridement + MFAT, Lipogems, 6–8 ml Arthroscopic debridement	36/31	//	//	III, IV	24
Wu (2023) (24)	Arthroscopic debridement + MFAT, Lipogems Arthroscopic debridement + HA, HA, 5 ml, 3 injections, 4 weeks apart	146/146	95/101	56.4/54.8	II, III	24
Zaffagnini (2022) (25)	MFAT, Lipogems, 5 ml LR-PRP, Manual, 5 ml	53/55	25/19	54.5/54.1	I, II, III, IV	24

^a^K-L grade, Kellgren-Lawrence grade for classifying OA severity, ranging from 0 (no OA) to IV (severe OA); ^b^//, missing data; HA, hyaluronic acid; LP, leukocyte-poor; LR, leukocyte-rich; PRP, platelet-rich plasma; MFAT, microfragmented adipose tissue.

### Risk-of-bias assessment

**Figure 1:**
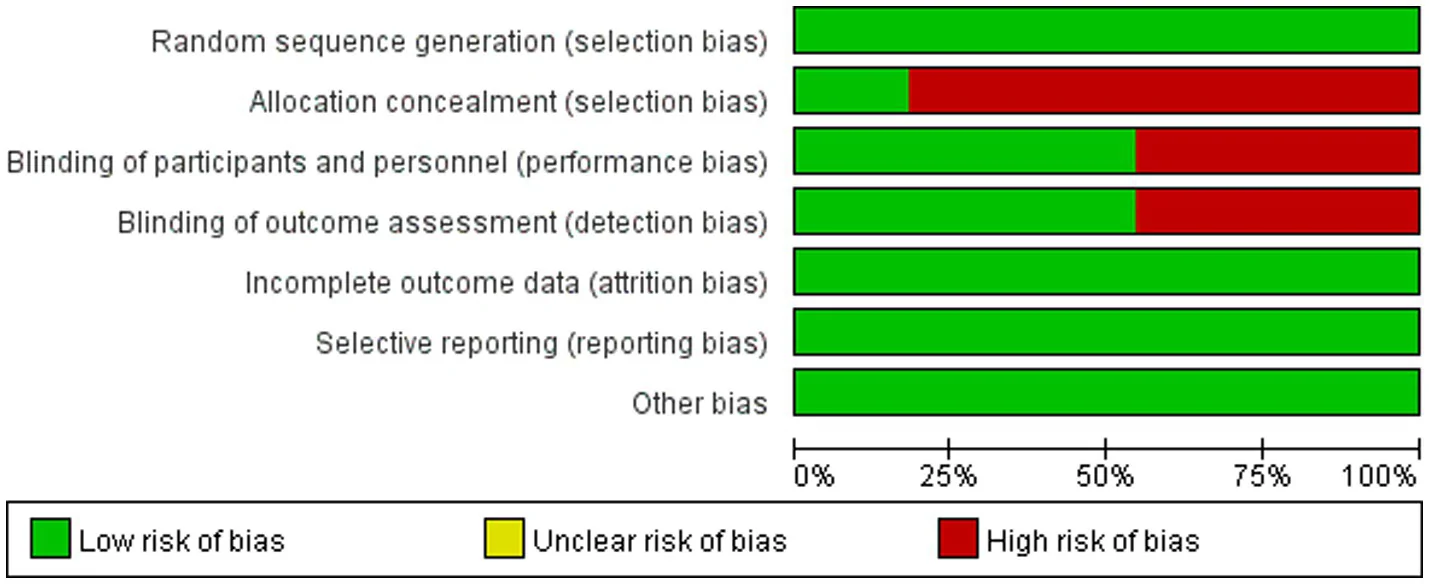
Risk of bias graph: review authors’ judgements about each risk of bias item presented as percentages across all included studies.

All included studies were randomized controlled trials with a generally low risk of bias across key domains. While all reported adequate random sequence generation, allocation concealment was not feasible for the MFAT intervention group relative to the comparator arms (PRP, HA, and saline) because of procedural constraints inherent to autologous tissue processing, which precluded allocation blinding at the point of randomization. Consequently, most studies were rated as having “high risk” regarding allocation concealment. Blinding of participants and outcome assessors was not reported in five studies. The full risk-of-bias assessment for each study is presented in Figure 1 and Supplementary Figure 2.

### Pain improvement

#### VAS score

**Figure 2:**
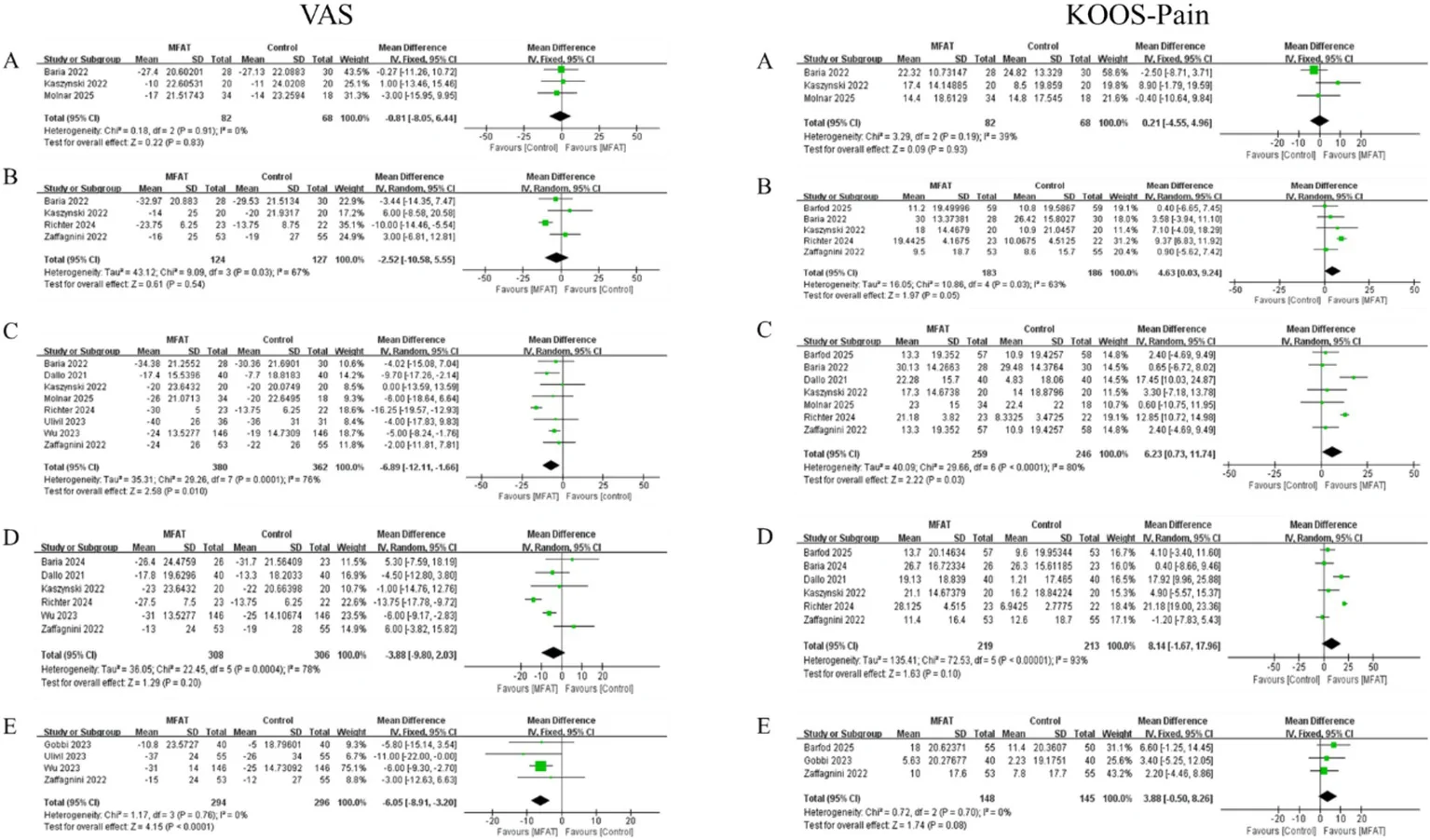
Forest plots of improvement in VAS and KOOS-Pain at 1 **(A)**, 3 **(B)**, 6 **(C)**, 12 **(D)**, and 24 **(E)** months detailing mean differences and 95% CIs comparing intra-articular injection of MFAT with Controls. IV, inverse variance; CI, confidence interval; KOOS, Knee injury and Osteoarthritis Outcome Score; VAS, visual analog scale; MFAT, microfragmented adipose tissue.

The pooled clinical outcomes for pain relief, as measured by the visual analog scale (VAS), across all included studies are summarized in Figure 2. For 1-, 3- and 12-month post-intervention data, three (17, 20, 21), four (17, 20, 22, 25) and six (16, 18, 20, 22, 24, 25) studies, respectively, contributed to the meta-analysis. No statistically significant difference in VAS score was observed between the MFAT group and the control group at any time point (1 month: MD = −0.81; 95% CI, −8.05 to 6.44; *p* = 0.83; I^2^ = 0%; 3 months: MD = −2.52; 95% CI, −10.58 to 5.55; *p* = 0.54; I^2^ = 67%; 12 months: MD = −3.88; 95% CI, −9.80 to 2.03; *p* = 0.20; I^2^ = 78%). In contrast, at follow-up intervals of 6 and 24 months, eight (17, 18, 20–25) and four (19, 23–25) studies, respectively, were available for analysis. Consistent and statistically significant reductions in VAS scores favored the MFAT group across both time points (6 months: MD = −6.89; 95% CI, −12.11 to −1.66; *p* = 0.01; I^2^ = 76%; 24 months: MD = −5.88; 95% CI, −8.79 to −2.97; *p* < 0.0001; I^2^ = 0%).

**Figure 3:**
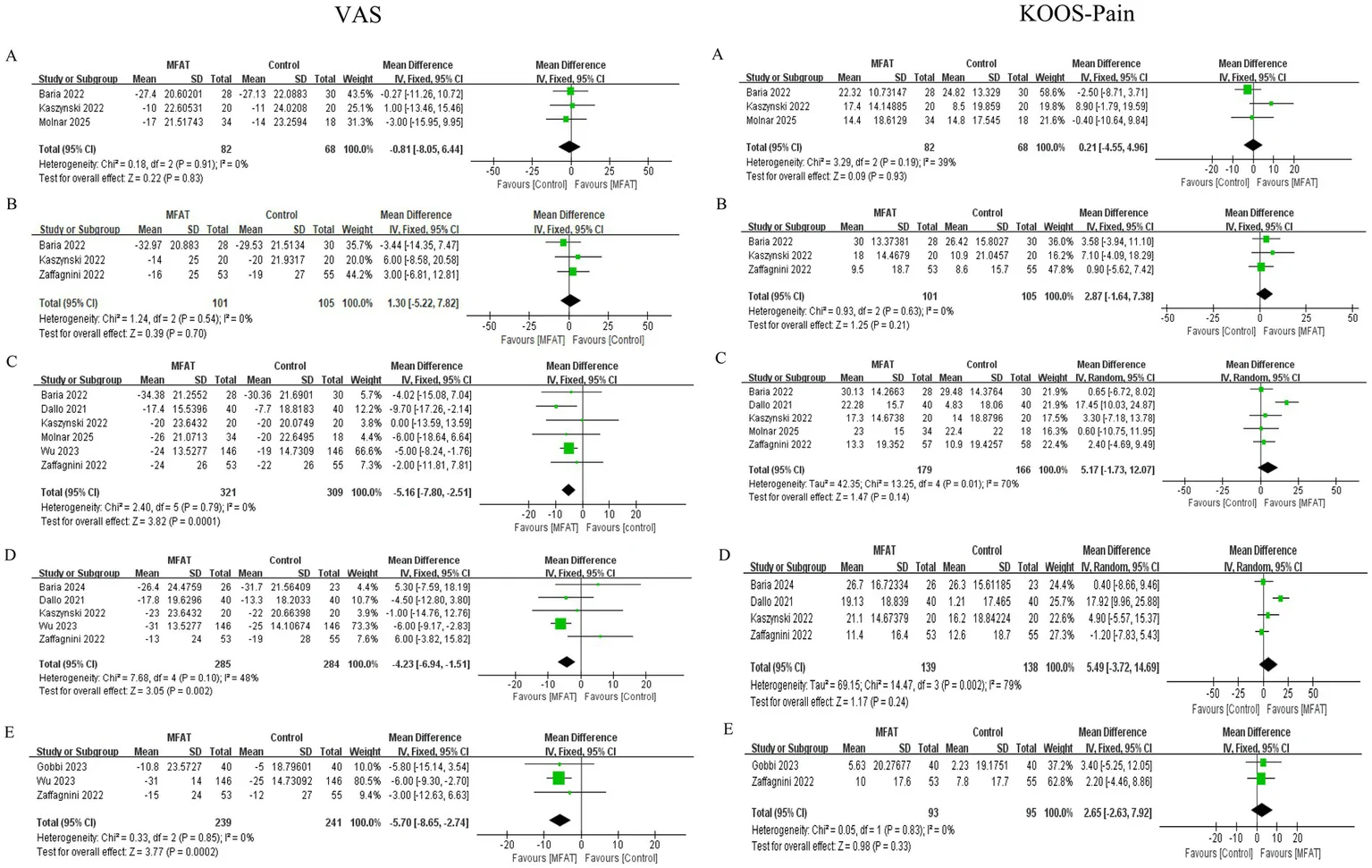
Forest plots of improvement in VAS and KOOS-Pain at 1 **(A)**, 3 **(B)**, 6 **(C)**, 12 **(D)**, and 24 **(E)** months detailing mean differences and 95% CIs comparing intra-articular injection of MFAT with PRP and HA. IV, inverse variance; CI, confidence interval; KOOS, Knee injury and Osteoarthritis Outcome Score; VAS, visual analog scale; MFAT, microfragmented adipose tissue; PRP, platelet-rich plasma; HA, hyaluronic acid.

As three studies (15, 22, 23) lacked a biologic-based positive control group, we excluded them to enable a more precise comparison of MFAT versus other biologics and performed a prespecified subgroup meta-analysis using the remaining studies. Three studies reported 1- and 3-month VAS data; pooled estimates demonstrated no statistically significant difference between MFAT and the comparator biologics (1 month: MD = −0.81; 95% CI, −8.05 to 6.44; *p* = 0.83; I^2^ = 0%; 3 months: MD = 1.30; 95% CI, −5.22 to 7.82; *p* = 0.70; I^2^ = 0%). For 6-, 12-, and 24-month data, six, five, and three studies were included, respectively; the analysis consistently favored MFAT across all time points (6 months: MD = −5.16; 95% CI, −7.80 to −2.51; *p* = 0.0001; I^2^ = 0%; 12 months: MD = −4.23; 95% CI, −6.94 to −1.51; *p* = 0.002; I^2^ = 48%; 24 months: MD = −5.70; 95% CI, −8.65 to −2.74; *p* = 0.0002; I^2^ = 0) (Figure 3).

#### KOOS pain

The pooled clinical outcomes for pain-specific knee function, as measured by the KOOS pain subscale, across all included studies are summarized in Figure 2. Three studies (17, 20, 21) contributed 1-month data; the pooled estimate indicated no statistically significant difference between the MFAT group and control groups (MD = 0.21; 95% CI, −4.55 to 4.96; *p* = 0.93; I^2^ = 39%). For 3- and 6-month data, five (15, 17, 20, 22, 25) and seven (15, 17, 18, 20–22, 25) studies, respectively, were available for analysis; compared with controls, MFAT was associated with statistically significant improvements in the KOOS pain score (3 months: MD = 4.63; 95% CI, 0.03 to 9.24; *p* = 0.05; I^2^ = 63%; 6 months: MD = 6.23; 95% CI, 0.73 to 11.74; *p* = 0.03; I^2^ = 80%). For 12- and 24-month data, six (15, 16, 18, 20, 22, 25) and three studies (15, 19, 25), respectively, were included; however, no statistically significant differences were observed (12 months: MD = 8.14; 95% CI, −1.67 to 17.96; *p* = 0.10; I^2^ = 93%; 24 months: MD = 3.88; 95% CI, −0.50 to 8.26; *p* = 0.08; I^2^ = 0%).

As three studies (15, 22, 23) lacked a biologic-based positive control group, we excluded them to enable a more precise comparison of MFAT versus other biologics and performed a prespecified subgroup meta-analysis using the remaining studies. KOOS pain scores were analyzed at five time points: 1 month (*n* = 3 studies), 3 months (*n* = 3), 6 months (*n* = 5), 12 months (*n* = 4), and 24 months (*n* = 2). Pooled estimates revealed no statistically significant differences between MFAT and comparator biologics at any follow-up interval (1 month: MD = 0.21; 95% CI, −4.55 to 4.96; *p* = 0.93; I^2^ = 39%; 3 months: MD = 2.87; 95% CI, −1.64 to 7.38; *p* = 0.21; I^2^ = 0%; 6 months: MD = 5.17; 95% CI, −1.73 to 12.07; *p* = 0.14; I^2^ = 70%; 12 months: MD = 5.49; 95% CI, −3.72 to 14.69; *p* = 0.24; I^2^ = 79%; 24 months: MD = 2.65; 95% CI, −2.63 to 7.92; *p* = 0.33; I^2^ = 0%) (Figure 3).

### Functional improvement

#### KOOS symptoms

**Figure 4:**
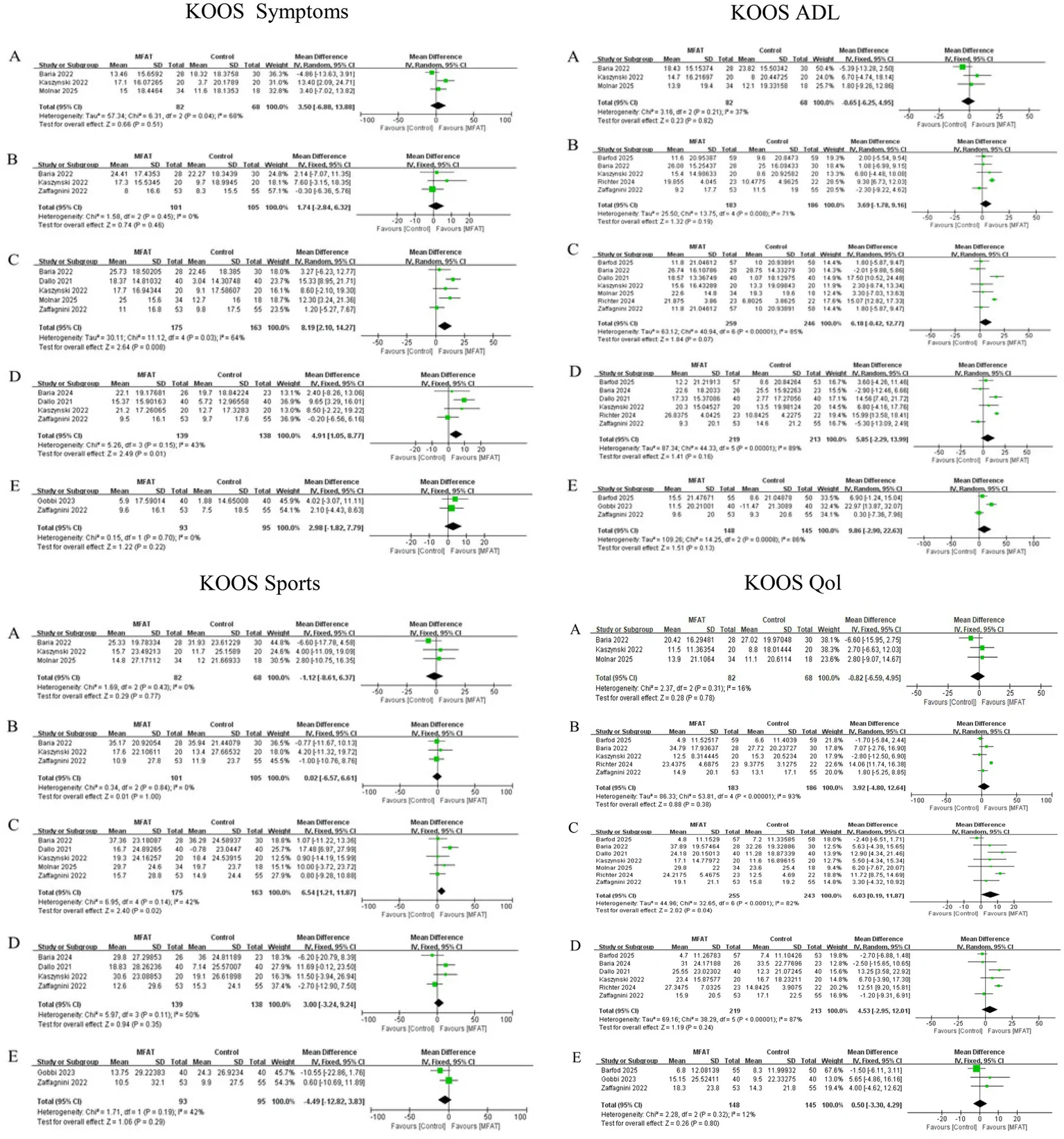
Forest plots of improvement in KOOS-Symptoms, KOOS-ADL, KOOS-Sports and KOOS-QoLat 1 **(A)**, 3 **(B)**, 6 **(C)**, 12 **(D)**, and 24 **(E)** months detailing mean differences and 95% CIs comparing intra-articular injection of MFAT with Controls. IV, inverse variance; CI, confidence interval; KOOS, Knee injury and Osteoarthritis Outcome Score; ADL, Activity of Daily Living; QoL, Quality of Life; MFAT, microfragmented adipose tissue.

The pooled clinical outcomes for knee-related symptoms, as measured by the KOOS symptoms subscale, across all included studies are summarized in Figure 4. All control groups in these studies consisted of biologic agents—ensuring a homogeneous comparator context for evaluating MFAT. Three studies contributed 1- (17, 20, 21) and 3-month (17, 20, 25) data; pooled estimates revealed no statistically significant difference between MFAT and other biologics (1 month: MD = 3.50; 95% CI, −6.88 to 13.88; *p* = 0.51; I^2^ = 68%; 3 months: MD = 1.74; 95% CI, −2.84 to 6.32; *p* = 0.46; I^2^ = 0%). For 6- and 12-month data, five (17, 18, 20, 21, 25) and four (16, 18, 20, 25) studies, respectively, were available for analysis; relative to comparators, MFAT demonstrated statistically significant improvements in symptom scores (6 months: MD = 8.19; 95% CI, 2.10 to 14.27; *p* = 0.008; I^2^ = 64%; 12 months: MD = 4.91; 95% CI, 1.05 to 8.77; *p* = 0.01; I^2^ = 43%). Two studies (19, 25) provided 24-month data; the pooled estimate was not significantly different (MD = 2.98; 95% CI, −1.82 to 7.79; *p* = 0.22; I^2^ = 0%).

#### KOOS ADL

The pooled clinical outcomes for activities of daily living, as measured by the KOOS ADL, across all included studies are summarized in Figure 4. KOOS ADL were analyzed at five time points: 1 month [*n* = 3 studies (17, 20, 21)], 3 months [*n* = 5 (15, 17, 20, 22, 25)], 6 months [*n* = 7 (15, 17, 18, 20–22, 25)], 12 months [*n* = 6 (15, 16, 18, 20, 22, 25)], and 24 months [*n* = 3 (15, 19, 25)]. Pooled estimates revealed no statistically significant differences between MFAT and comparator biologics at any follow-up interval (1 month: MD = −0.65; 95% CI, −6.25 to 4.95; *p* = 0.82; I^2^ = 37%; 3 months: MD = 3.69; 95% CI, −1.78 to 9.16; *p* = 0.19; I^2^ = 71%; 6 months: MD = 6.18; 95% CI, −0.42 to 12.77; *p* = 0.07; I^2^ = 85%; 12 months: MD = 5.85; 95% CI, −2.29 to 13.99; *p* = 0.16; I^2^ = 89%; 24 months: MD = 9.86; 95% CI, −2.90 to 22.63; *p* = 0.13; I^2^ = 86%).

**Figure 5:**
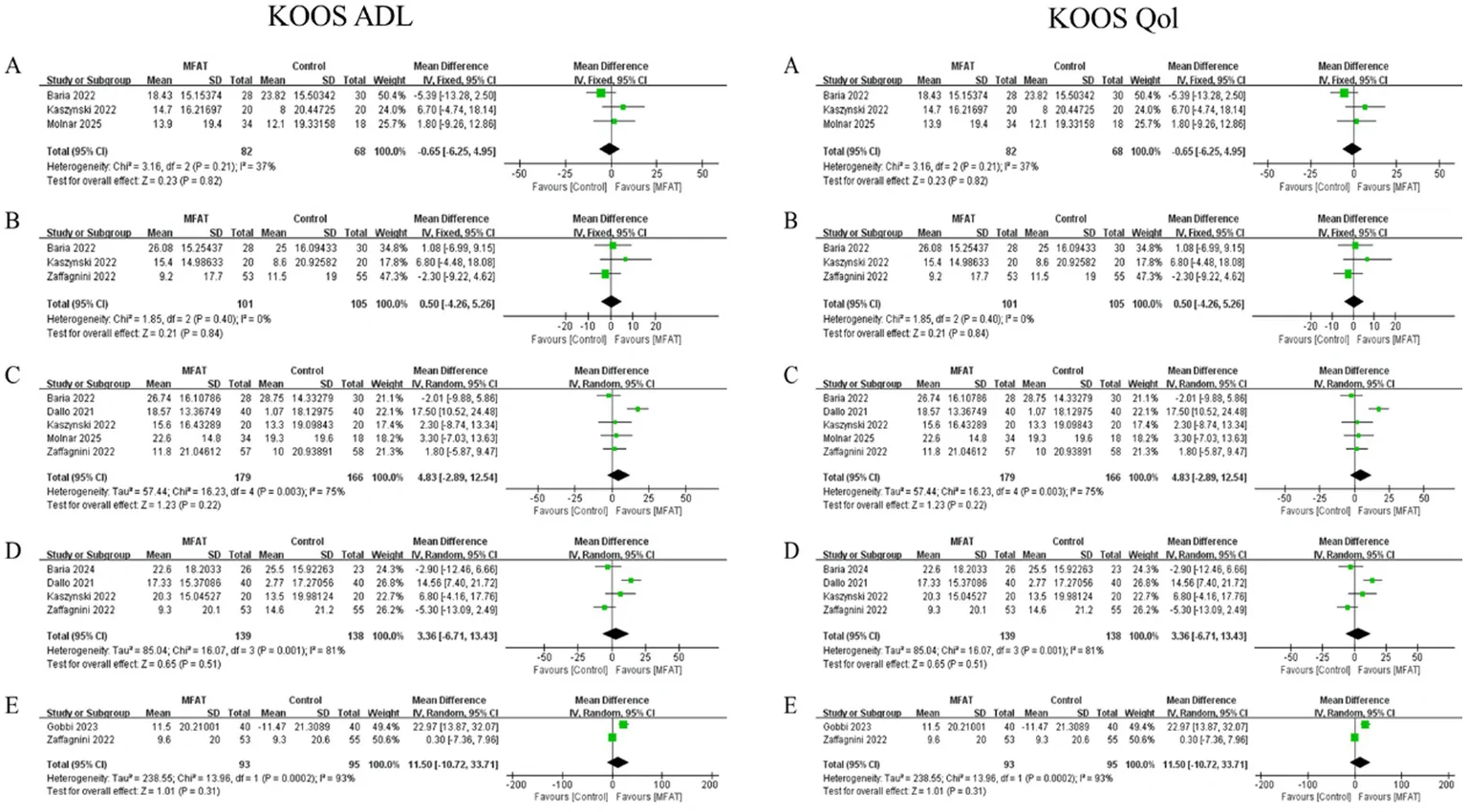
Forest plots of improvement in KOOS-ADL and KOOS-QoLat 1 **(A)**, 3 **(B)**, 6 **(C)**, 12 **(D)**, and 24 **(E)** months detailing mean differences and 95% CIs comparing intra-articular injection of MFAT with PRP and HA. IV, inverse variance; CI, confidence interval; KOOS, Knee injury and Osteoarthritis Outcome Score; ADL, Activity of Daily Living; QoL, Quality of Life; MFAT, microfragmented adipose tissue; PRP, platelet-rich plasma; HA, hyaluronic acid.

To refine the comparative assessment of MFAT versus other biologics, we excluded three studies (15, 22, 23) lacking a biologic-based control group and performed a prespecified subgroup meta-analysis using the remaining studies. KOOS ADL scores were analyzed at five time points: 1 month (*n* = 3 studies), 3 months (*n* = 3), 6 months (*n* = 5), 12 months (*n* = 4), and 24 months (*n* = 2). Pooled estimates revealed no statistically significant differences between MFAT and comparator biologics at any follow-up interval (1 month: MD = −0.65; 95% CI, −6.25 to 4.95; *p* = 0.82; I^2^ = 37%; 3 months: MD = 0.50; 95% CI, −4.26 to 5.26; *p* = 0.84; I^2^ = 0%; 6 months: MD = 4.83; 95% CI, −2.89 to 12.54; *p* = 0.22; I^2^ = 75%; 12 months: MD = 3.36; 95% CI, −6.71 to 13.43; *p* = 0.51; I^2^ = 71%; 24 months: MD = 11.50; 95% CI, −10.72 to 33.71; *p* = 0.31; I^2^ = 93%) (Figure 5).

#### KOOS sport

The pooled clinical outcomes for sport and recreation function, as measured by the KOOS sport, across all included studies are summarized in Figure 4. All control groups in these studies consisted of biologic agents—ensuring a homogeneous comparator context for evaluating MFAT. Three studies contributed 1- (17, 20, 21) and 3-month (17, 20, 25) data; pooled estimates revealed no statistically significant difference between MFAT and other biologics (1 month: MD = −1.12; 95% CI, −8.61 to 6.37; *p* = 0.77; I^2^ = 0%; 3 months: MD = 0.02; 95% CI, −6.57 to 6.61; *p* = 1.00; I^2^ = 0%). For 6-month data, five studies (17, 18, 20, 21, 25) were available for analysis; relative to comparators, MFAT demonstrated statistically significant improvements in KOOS sport (MD = 6.54; 95% CI, 1.21 to 11.87; *p* = 0.02; I^2^ = 42%). Four (16, 18, 20, 25) and two studies (19, 25) provided 12- and 24-month data; the pooled estimate was not significantly different (12 months: MD = 3.00; 95% CI, −3.24 to 9.24; *p* = 0.35; I^2^ = 50%; 24 months: MD = −4.49; 95% CI, −12.82 to 3.83; *p* = 0.29; I^2^ = 42%).

#### KOOS QOL

The pooled clinical outcomes for knee-related quality of life, as measured by the KOOS QOL, across all included studies are summarized in Figure 4. KOOS QOL were analyzed at five time points: 1 month [*n* = 3 studies (17, 20, 21)], 3 months [*n* = 5 (15, 17, 20, 22, 25)], 6 months [*n* = 7 (15, 17, 18, 20–22, 25)], 12 months [*n* = 6 (15, 16, 18, 20, 22, 25)], and 24 months [*n* = 3 (15, 19, 25)]. Pooled estimates revealed no statistically significant differences between MFAT and comparator biologics at any follow-up interval (1 month: MD = −0.76; 95% CI, −7.07 to 5.55; *p* = 0.81; I^2^ = 16%, 3 months: MD = 3.92; 95% CI, −4.80 to 12.64; *p* = 0.38; I^2^ = 93%, 12 months: MD = 4.53; 95% CI, −2.95 to 12.01; *p* = 0.24; I^2^ = 85%, 24 months: MD = 0.50; 95% CI, −3.30 to 4.29; *p* = 0.80; I^2^ = 12%). At 6 months, however, MFAT demonstrated a statistically significant improvement in KOOS QOL relative to controls (MD = 6.03; 95% CI, 0.19 to 11.87; *p* = 0.04; I^2^ = 82%).

To refine the comparative assessment of MFAT versus other biologics, we excluded three studies (15, 22, 23) lacking a biologic-based control group and performed a prespecified subgroup meta-analysis using the remaining studies. KOOS QOL scores were analyzed at five time points: 1 month (*n* = 3 studies), 3 months (*n* = 3), 6 months (*n* = 5), 12 months (*n* = 4), and 24 months (*n* = 2). Pooled estimates revealed no statistically significant differences between MFAT and comparator biologics at 1 month (MD = −0.76; 95% CI, −7.07 to 5.55; p = 0.81; I^2^ = 16%) or 3 months (MD = 1.94; 95% CI, −2.99 to 6.87; *p* = 0.44; I^2^ = 0%). At 6 months, MFAT demonstrated a statistically significant and clinically relevant improvement in knee-related quality of life (MD = 6.71; 95% CI, 2.50 to 10.92; *p* = 0.002; I^2^ = 0%). No significant differences were observed at 12 months (MD = 4.27; 95% CI, −3.09 to 11.63; *p* = 0.26; I^2^ = 52%) or 24 months (MD = 4.66; 95% CI, −2.00 to 11.33; *p* = 0.17; I^2^ = 0%) (Figure 5).

#### Tegner

**Figure 6:**
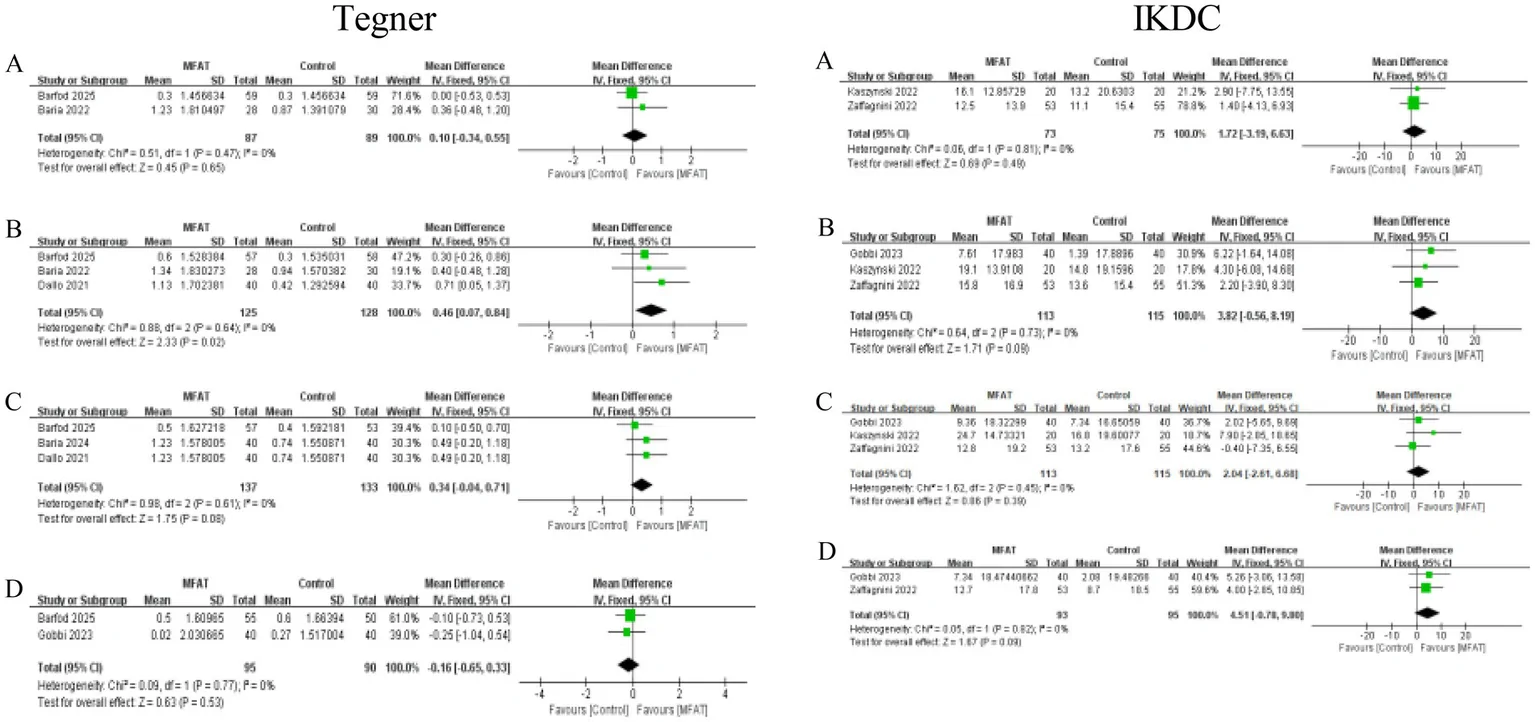
Forest plots of improvement in Tegner and IKDC subjective scores at 3 **(A)**, 6 **(B)**, 12 **(C)**, and 24 **(D)** months detailing mean differences and 95% CIs comparing intra-articular injection of MFAT with Controls. IV, inverse variance; CI, confidence interval; IKDC, International Knee Documentation Committee; MFAT, microfragmented adipose tissue.

The pooled clinical outcomes for functional activity level, as measured by the Tegner, across all included studies are summarized in Figure 6. Two studies (15, 17) contributed 3-month data; the pooled estimate indicated no statistically significant difference between the MFAT group and control groups (MD = 0.10; 95% CI, −0.34 to 0.55; *p* = 0.65; I^2^ = 0%). For 6-month data, three studies (15, 17, 18) were available for analysis; compared with controls, MFAT was associated with statistically significant improvements in the Tegner (MD = 0.46; 95% CI, 0.07 to 0.84; *p* = 0.02; I^2^ = 0%). For 12- and 24-month data, Three (15, 16, 18) and two studies (15, 19), respectively, were included; however, no statistically significant differences were observed (12 months: MD = 0.34; 95% CI, −0.04 to 0.71; *p* = 0.08; I^2^ = 0%; 24 months: MD = −0.16; 95% CI, −0.65 to 0.33; *p* = 0.53; I^2^ = 0%).

**Figure 7:**
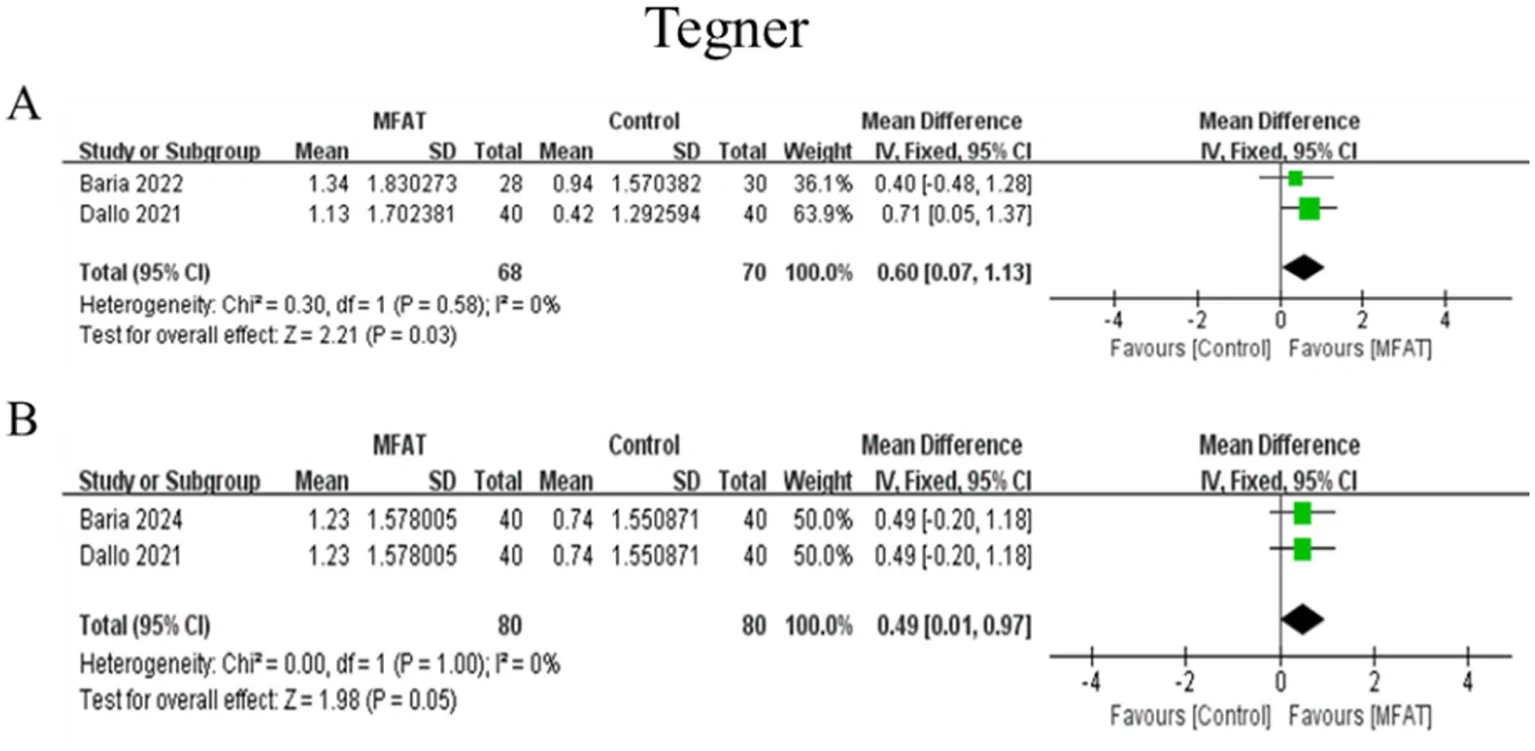
Forest plots of improvement in Tegner at 6 **(A)** and 12 **(B)** months detailing mean differences and 95% CIs comparing intra-articular injection of MFAT with PRP and HA. IV, inverse variance; CI, confidence interval; MFAT, microfragmented adipose tissue; PRP, platelet-rich plasma; HA, hyaluronic acid.

To refine the comparative assessment of MFAT versus other biologics, we excluded three studies (15, 22, 23) lacking a biologic-based control group and performed a prespecified subgroup meta-analysis using the remaining studies. For 6-, and 12-month data, two studies were included, respectively; the analysis consistently favored MFAT across both time points (MD = 0.60; 95% CI, 0.07 to 1.13; *p* = 0.03; I^2^ = 0%) and at 12 months (MD = 0.49; 95% CI, 0.01 to 0.97; *p* = 0.05; I^2^ = 0%) (Figure 7).

#### IKDC

The pooled clinical outcomes for knee-specific subjective function, as measured by the *IKDC*, across all included studies are summarized in Figure 6. All control groups in these studies consisted of biologic agents—ensuring a homogeneous comparator context for evaluating MFAT. IKDC were analyzed at four time points: 3 months [*n* = 2 studies (20, 25)], 6 months [*n* = 3 (19, 20, 25)], 12 months [*n* = 3 (19, 20, 25)], and 24 months [*n* = 2 (19, 25)]. Pooled estimates revealed no statistically significant differences between MFAT and comparator biologics at any follow-up interval (3 months: MD = 1.72; 95% CI, −3.19 to 6.63; *p* = 0.49; I^2^ = 0%; 6 months: MD = 3.82; 95% CI, −0.56 to 8.19; *p* = 0.09; I^2^ = 0%; 12 months: MD = 2.04; 95% CI, −2.61 to 6.68; *p* = 0.39; I^2^ = 0%; 24 months: MD = 4.51; 95% CI, −0.78 to 9.80; *p* = 0.09; I^2^ = 0%).

#### WOMAC

**Figure 8:**
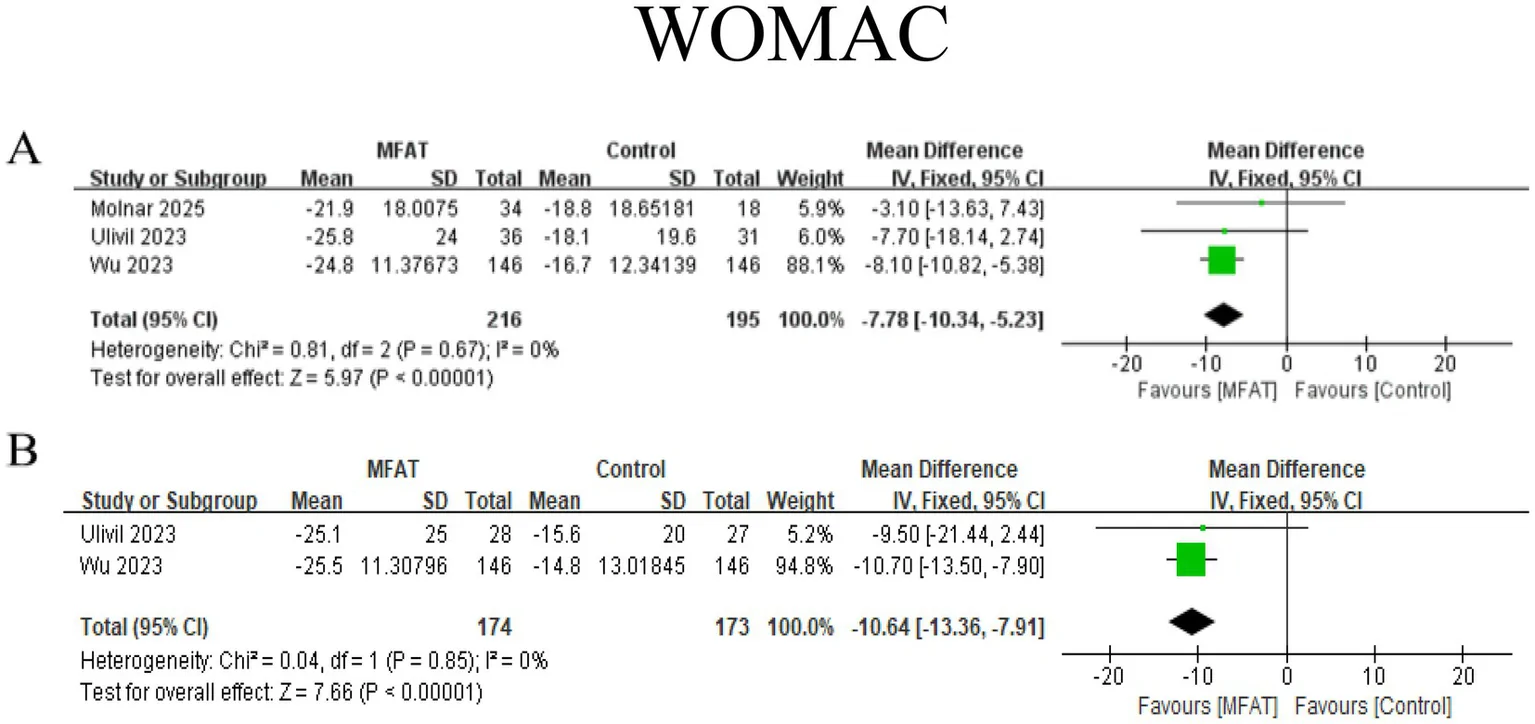
Forest plots of improvement in WOMAC scores at 6 **(A)** and 24 **(B)** months detailing mean differences and 95% CIs comparing intra-articular injection of MFAT with controls. IV, inverse variance; CI, confidence interval; WOMAC, Western Ontario and McMaster Universities Osteoarthritis Index; MFAT, microfragmented adipose tissue.

The pooled clinical outcomes for pain, stiffness, and physical function—assessed collectively by the WOMAC Index—across all included studies are summarized in Figure 8. For 6- and 24-month data, Three (21, 23, 24) and two (23, 24), respectively, were available for analysis; compared with controls, MFAT was associated with statistically significant improvements in the WOMAC scores (6 months: MD = −7.78; 95% CI, −10.34 to −5.23; *p* < 0.00001; I^2^ = 0%; 24 months: MD = −10.64; 95% CI, −13.36 to −7.91; p < 0.00001; I^2^ = 0%).

**Figure 9:**
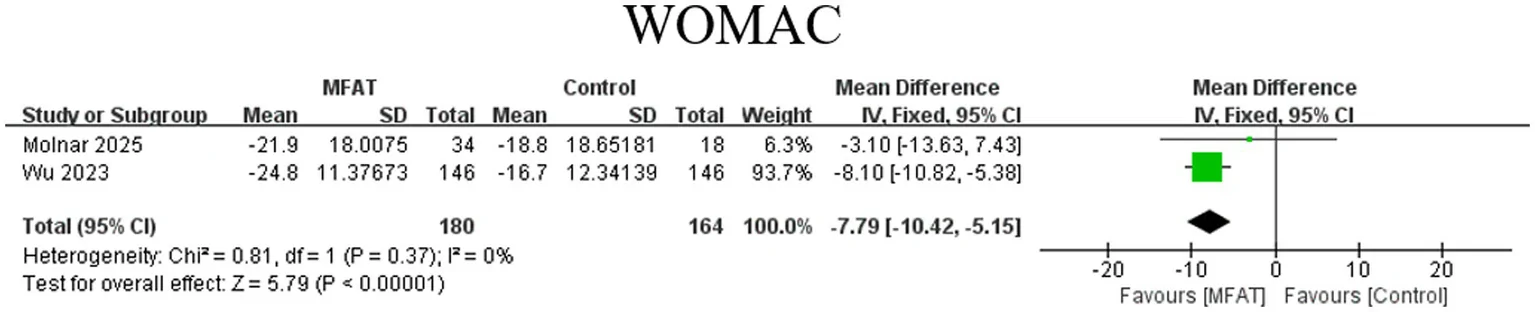
Forest plots of improvement in WOMAC scores at 6 months detailing mean differences and 95% CIs comparing intra-articular injection of MFAT with PRP and HA. IV, inverse variance; CI, confidence interval; WOMAC, Western Ontario and McMaster Universities Osteoarthritis Index; MFAT, microfragmented adipose tissue; PRP, platelet-rich plasma; HA, hyaluronic acid.

To refine the comparative assessment of MFAT versus other biologics, we excluded three studies (15, 22, 23) lacking a biologic-based control group and performed a prespecified subgroup meta-analysis using the remaining studies. For 6- month data, two studies were available for analysis; relative to comparators, MFAT demonstrated statistically significant improvements in WOMAC scores (MD = −7.79; 95% CI, −10.42 to −5.15; *p* < 0.00001; I^2^ = 0%) (Figure 9).

#### Safety

Six studies reported treatment-related adverse events. Two studies (17, 18) reported zero treatment-related adverse events. A pooled analysis of the remaining four studies (19, 23–25) revealed no statistically significant difference in the incidence of treatment-related adverse events between the MFAT group and control groups (RR = 0.85; 95% CI, 0.51 to 1.43; *p* = 0.55; I^2^ = 26%) (Supplementary Figure 3). Among these, three studies (19, 24, 25) specifically compared MFAT with other biologic agents; their pooled estimate similarly showed no significant difference (RR = 0.81; 95% CI, 0.48 to 1.38; *p* = 0.44; I^2^ = 41%) (Supplementary Figure 4). All reported treatment-related adverse events—including injection-site pain and/or swelling—were transient and resolved spontaneously within a few days without clinical intervention.

## Discussion

A prior systematic review—incorporating heterogeneous study designs—reported that intra-articular MFAT injection significantly improved pain and functional outcomes in patients with knee osteoarthritis, with improvements in select endpoints surpassing established MCID thresholds (26). A subsequent systematic review by Park et al. corroborated these findings (27). Hohmann et al. reported null results in their comparative analysis of MFAT versus other orthopedic biologics; however, this conclusion was based on a mixed-evidence pool that included nonrandomized studies, compromising internal validity—the authors themselves cautioned against overinterpretation (28). To address this limitation, the present meta-analysis applied stringent eligibility criteria, restricting inclusion to high-quality evidence (level I and II studies only). Our results demonstrate that at both 6- and 12-month follow-ups, compared with PRP, HA, and saline, MFAT yielded statistically significant advantages in VAS pain scores, KOOS scores, and WOMAC total scores (6- and 24-month). However, these statistical differences should be interpreted with caution, as the magnitude of improvement did not reach established MCID thresholds across all pooled analyses. Moreover, the observed superiority was primarily driven by core symptom endpoints (VAS and KOOS symptom), whereas distal functional measures such as IKDC and KOOS-ADL showed no significant between-group differences, suggesting that MFAT’s therapeutic signal may be domain-specific rather than generalized. No significant differences in treatment-related adverse event incidence were observed between MFAT and controls, reinforcing its favorable safety profile.

Intra-articular injection therapy has long been a central strategy in OA management. HA is endorsed as a first-line treatment by multiple international guidelines (2, 29–31). Network meta-analyses consistently rank PRP above other injectables in terms of efficacy (32, 33). Saline injection alone yields modest yet reproducible improvements in pain and function (34, 35)—effects attributable to placebo mechanisms—and remains the methodologically appropriate placebo control. To ensure a rigorous benchmark, we pooled all guideline-endorsed comparators—HA, PRP, and saline—into a unified control group. To specifically evaluate MFAT against active biologics, we performed a prespecified subgroup analysis excluding saline-controlled trials. At both follow-ups, MFAT demonstrated statistically significant superiority over PRP and HA. However, as in the primary analysis, the effect sizes remained below MCID thresholds, indicating that even when benchmarked exclusively against active biologics, statistical advantages do not translate into clinically meaningful differences at the patient level. Heterogeneity was markedly reduced in this subgroup analysis, indicating enhanced consistency and precision. Nevertheless, this sub-MCID pattern—statistical consistency without clinical significance—suggests that MFAT may exert a genuine but modest biological effect that, at least within the 12-month window, does not yield patient-perceptible improvements.

Microfragmentation is a single-step technique designed to preserve the native architecture and extracellular matrix of adipose tissue (36, 37), maintaining the viability and paracrine functionality of resident MSCs. Adipose tissue contains a 10,000-fold greater density of reparative cells compared with peripheral blood (38), establishing a biological rationale for MFAT’s enhanced performance relative to MSC-deficient PRP. Nevertheless, several factors may explain the translational gap between this robust rationale and the sub-MCID effect sizes observed. The paracrine activity of MFAT-derived MSCs may be attenuated in the inflammatory OA joint environment; the delivered extracellular matrix may impede rapid diffusion of secreted factors; and preparation variability across studies may dilute aggregate effect sizes. Clinical MRI-based studies by Molnar et al. and Boric et al. demonstrated that MFAT increased intracartilaginous GAG content and provided imaging evidence of enhanced chondrocyte anabolic activity (21, 39). These structural improvements, while encouraging as proof-of-concept, have not yet been shown to correlate with patient-reported outcomes crossing the MCID threshold—highlighting the distinction between surrogate and clinically meaningful endpoints. Importantly, this discrepancy between statistical significance and clinical relevance is not unique to MFAT; it mirrors the experience with PRP, where early meta-analyses similarly showed statistical signals that preceded—but did not guarantee—subsequent confirmation of clinical efficacy in longer-term trials. At present, several comparative trials reported nonsignificant differences, potentially attributable to limited sample sizes and elevated type II error risk. By synthesizing high-quality RCTs, we substantially increased statistical power to obtain more precise estimates.

From a clinical standpoint, the sub-MCID findings do not render MFAT devoid of potential, but they do mandate a tempered positioning. MFAT should not be regarded as a first-line therapy, particularly given that PRP and HA have practical advantages: they require no liposuction, involve no specialized training, incur lower costs, and permit flexible repeat dosing (40). Instead, MFAT may be better considered as a second-line or adjunctive option for patients who have failed standard therapies, or for those with specific baseline characteristics—younger age, milder radiographic severity—that may render them more biologically responsive. Moreover, the absence of clinical significance at 12 months does not exclude cumulative or delayed benefits beyond this point; MRI-based evidence of structural effects (39), together with other long-term clinical studies reporting sustained improvements up to 24 months or beyond (41, 42), suggests that disease-modifying benefits may require observation windows exceeding 24 months to manifest as clinically perceptible improvements. Thus, MFAT’s regenerative potential should be weighed against practical considerations—balancing mechanistic promise with real-world feasibility and patient-centered value—while awaiting definitive evidence from adequately powered, long-term trials.

This study has several methodological limitations that warrant careful interpretation of the findings. First, the literature search was restricted to major English-language databases and excluded non-English publications, potentially introducing language bias and omitting high-quality studies published in other languages. Moreover, because fewer than 10 randomized trials were included in the final outcomes—falling below the widely accepted minimum threshold for the reliable detection of publication bias—Egger’s test and funnel plot asymmetry assessment were not conducted. This omission is fully aligned with the Cochrane and PRISMA recommendations for meta-analyses with limited study numbers and reflects deliberate methodological adherence, not analytical deficiency. Second, the available evidence is predominantly short- to medium-term: follow-up durations ranged from 6 to 12 months, which is inadequate for assessing long-term structural outcomes—including cartilage volume preservation, radiographic joint space narrowing progression, and delayed adverse events—associated with regenerative biologics such as PRP and MFAT. Consequently, rigorously designed, adequately powered RCTs featuring follow-up periods of more than 24 months are critically needed to establish whether these interventions yield sustained symptomatic improvement, true disease-modifying effects, or as-yet-unrecognized late-emergent safety issues. Third, despite our best efforts to minimize bias, substantial heterogeneity persisted across the included studies—stemming from patient variability, procedural differences, and comparator diversity. Some heterogeneity may thus reflect comparator differences rather than MFAT’s true effect. Although subgroup analyses partially compensated for this limitation, the pooled results should still be interpreted with caution. Moreover, owing to inconsistent reporting and unavailable individual participant data, we could not perform further subgroup or meta-regression analyses. Nevertheless, our findings provide the available evidence synthesis and offer valuable directional signals for future research.

## Conclusion

At 6 and 12 months, MFAT showed statistically significant improvements in pain (VAS) and core functional outcomes (KOOS symptom, WOMAC (6- and 24-month)) versus controls (PRP, HA, or saline), while IKDC, KOOS-ADL and KOOS-Sport demonstrated no significant between-group differences. All pooled effect sizes failed to reach MCID, confirming that statistical signals have not translated into clinical meaningfulness within 12 months. AEs were comparable to controls, supporting a favorable safety profile. Current evidence does not support MFAT as first-line therapy, but consistent statistical signals in core symptom endpoints, combined with its distinct structural modification mechanism, justify continued investigation. Definitive confirmation requires large-scale RCTs with follow-up exceeding 24 months and prespecified subgroup analyses (OA severity, BMI, baseline function) to define optimal target populations and generate practice-changing evidence.
