# Supplementary material for: Efficacy and safety of microfragmented adipose tissue versus platelet-rich plasma, hyaluronic acid and saline for knee osteoarthritis
Source: Front Med (Lausanne). 2026 Aug 11;13:1901713. doi: 10.3389/fmed.2026.1901713 (PMC13504197; doi:10.3389/fmed.2026.1901713)
Supplement: Supplementary file 1 [file Data_sheet_1.docx]

**Supplemental Fig. 1** Preferred Reporting Items for Systematic Reviews and Meta-Analyses (PRISMA) study selection flow diagram.

**Supplemental Fig. 2** Risk of bias summary: review authors' judgements about each risk of bias item for each included study

**Supplemental Fig. 3.** Forrest plot of Treatment-related adverse events detailing relative risk and 95% CIs comparing intra-articular injection of MFAT with Controls. M-H, Mantel-Haenszel; CI, confidence interval.

**Supplemental Fig. 4.** Forrest plot of Treatment-related adverse events detailing relative risk and 95% CIs comparing intra-articular injection of MFAT with PRP and HA. M-H, Mantel-Haenszel; CI, confidence interval; PRP, platelet-rich plasma; HA, hyaluronic acid.
